# Culture‐Negative Infective Endocarditis due to *Neisseria bacilliformis* : A Rare Case With Multisystem Embolization and Diagnostic Utility of Microbial Cell‐Free DNA Testing

**DOI:** 10.1002/ccr3.73099

**Published:** 2026-07-03

**Authors:** Hareem Saleem, Syed Muhammad Salman Hassan, Bilal Amjad, Esha Umair, Anum Faiz, Tayyaba Saleem, Muhammad Saaim, Ian Vargas, Moosa Mubarika, Lima Dalil, Muneeb Khawar

**Affiliations:** ^1^ Memorial Healthcare System Hollywood Florida USA; ^2^ Nishtar Medical University Multan Pakistan; ^3^ Jinnah Hospital Lahore Pakistan; ^4^ Rawalpindi Medical University Rawalpindi Pakistan; ^5^ Kabul Medical University Kabul Afghanistan; ^6^ King Edward Medical University Lahore Pakistan

**Keywords:** culture‐negative endocarditis, embolism, infective endocarditis, microbial cell‐free DNA, *Neisseria bacilliformis*

## Abstract

*Neisseria bacilliformis* is a rare cause of culture‐negative infective endocarditis with high embolic potential. Plasma microbial cell‐free DNA testing is crucial for diagnosis in blood culture‐negative cases, enabling targeted antibiotic therapy and improved outcomes despite multisystem complications.

## Graphical abstract

*Neisseria bacilliformis* , a rare cause of culture‐negative infective endocarditis, led to multisystem embolization (spleen, kidney, brain, lung) in a 79‐year‐old woman. Plasma microbial cell‐free DNA testing enabled definitive diagnosis and successful targeted therapy.

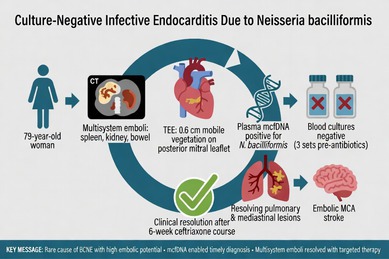

## 1 Introduction

A potentially fatal condition, infectious endocarditis (IE) has a significant morbidity and fatality rate, especially if the causing organism is unknown or the diagnosis is delayed [1, 2]. The diagnostic yield of traditional blood cultures is limited by blood culture negative infective endocarditis (BCNE), which accounts for a significant percentage of patients and is typically linked to previous antibiotic exposure or infection with fastidious bacteria [3].

The typical oropharyngeal microbiota includes the non‐gonococcal, non‐meningococcal species *Neisseria bacilliformis* . Despite being historically thought to have little pathogenic potential, this bacterium has been linked to an increasing number of invasive illnesses, such as bacteremia and infective endocarditis [4]. Because *N. bacilliformis* associated IE is uncommon and hard to find with conventional microbiological techniques, it is probably underdiagnosed. Rare fastidious organisms are less known causes of BCNE, often leading to delayed diagnosis and increased risk of embolic complications.

Recent advancements in molecular diagnostic methodologies, including plasma microbial cell‐free DNA testing, have enhanced the capacity to detect pathogens in BCNE, especially after the commencement of antibiotic therapy [4]. We provide a case of culture‐negative infective endocarditis attributed to *N. bacilliformis* , worsened by systemic embolization, underscoring the diagnostic significance of genetic testing and the growing clinical importance of this rare pathogen.

## 2 Case History/Examination

A 79‐year‐old woman with a history of hypertension and a recently treated urinary tract infection presented to the emergency department on January 11, 2026, with several days of abdominal pain accompanied by nausea and vomiting following the ingestion of crab. On initial evaluation, she was afebrile and hemodynamically stable, with no focal neurological deficits.

Laboratory studies demonstrated significant leukocytosis, with a white blood cell count of 21.6 × 10^9^/L, and an elevated C‐reactive protein level of 11.2 mg/dL; the erythrocyte sedimentation rate was within normal limits. Computed tomography of the abdomen and pelvis revealed acute infarctions involving the bowel, spleen (Figure 1), and kidney (Figure 2), raising concern for a systemic embolic process. Empiric broad‐spectrum antimicrobial therapy with piperacillin–tazobactam was initiated.

**FIGURE 1:**
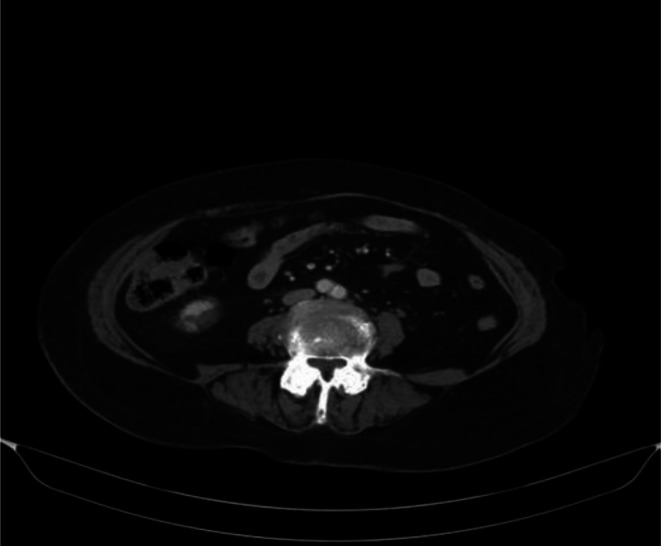
CT abdomen/pelvis (A/P) reveals a wedge‐shaped infarction in the spleen.

**FIGURE 2:**
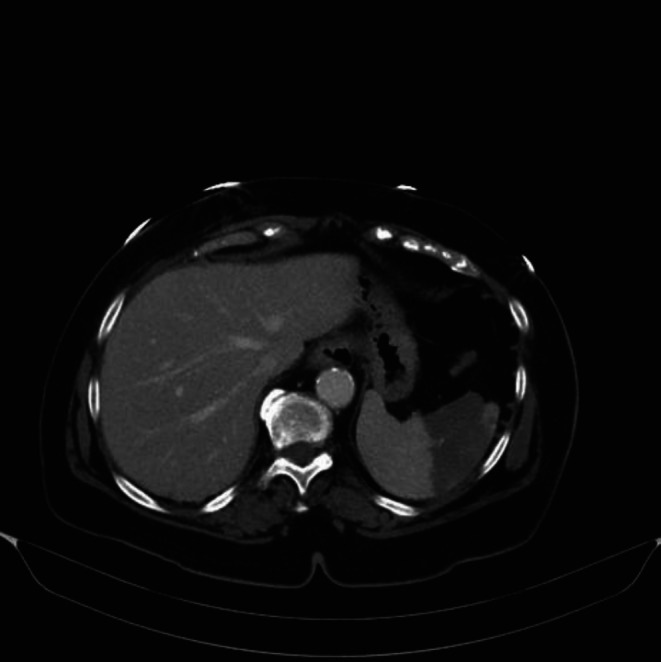
CT abdomen/pelvis (A/P) shows a wedge‐shaped infarction in the right kidney.

## 3 Differential Diagnosis, Investigations, and Treatment

The differential diagnosis for the patient's presentation with multisystem embolization involving the bowel, spleen, and kidney infarctions along with anterior mediastinal and pulmonary lesions included septic embolic phenomenon secondary to infective endocarditis as the most likely etiology given subsequent cardiac findings, metastatic malignancy, and other possible embolic sources such as cardiac thrombus or tumor emboli. Further evaluation with computed tomography angiography identified two anterior mediastinal lesions measuring 1.0 and 1.2 cm, as well as a 1.9 × 1.8 cm mass in the right upper lobe of the lung concerning for metastatic malignancy versus septic embolic phenomenon. Given the evidence of multisystem embolization, a cardiac source was suspected. Transesophageal echocardiography performed on hospital day three demonstrated a 0.6 cm mobile echodensity on the posterior mitral valve leaflet, consistent with a vegetation (Figure 3).

**FIGURE 3:**
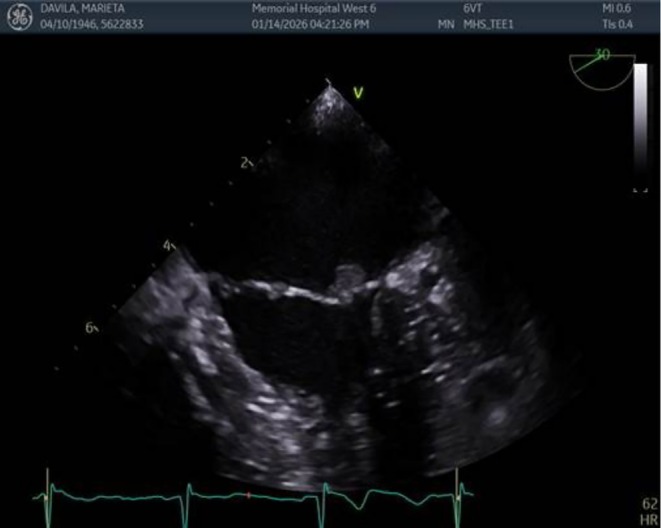
Transesophageal echocardiography (TEE) shows a mitral valve vegetation on the atrial side of the mitral valve (arrow).

Blood cultures obtained after initiation of antimicrobial therapy remained negative throughout the hospital course. Although the patient initially showed clinical improvement, she subsequently developed intermittent low‐grade fevers and fluctuating leukocytosis. Plasma microbial cell‐free DNA testing was therefore pursued and identified *Neisseria bacilliformis* , confirming the diagnosis of infective endocarditis due to this rare pathogen. Three sets of peripheral blood cultures were obtained from separate venipuncture sites prior to the administration of any antibiotics. Piperacillin–tazobactam was initiated approximately 4 h after blood culture collection on the day of admission. Additional blood culture sets were drawn on hospital days 2 and 4 while the patient was receiving antimicrobial therapy. All cultures remained negative after 5 days of incubation in both aerobic and anaerobic bottles.

Empiric intravenous broad‐spectrum antibiotics were initiated in accordance with guideline recommendations. Following identification of *N. bacilliformis* , ceftriaxone 2 g daily was continued to complete a six‐week course.

## 4 Outcome and Follow‐Up

During hospitalization, the patient developed an acute ischemic stroke. Magnetic resonance imaging (MRI) of the brain demonstrated a large ischemic stroke in the right middle cerebral artery (MCA) territory, along with smaller ischemic lesions in the bilateral frontal lobes and the left occipital lobe (Figure 4), consistent with embolic cerebrovascular disease. She also experienced an acute decline in hemoglobin to 6.5 g/dL, requiring transfusion of one unit of packed red blood cells, after which she reported symptomatic improvement.

**FIGURE 4:**
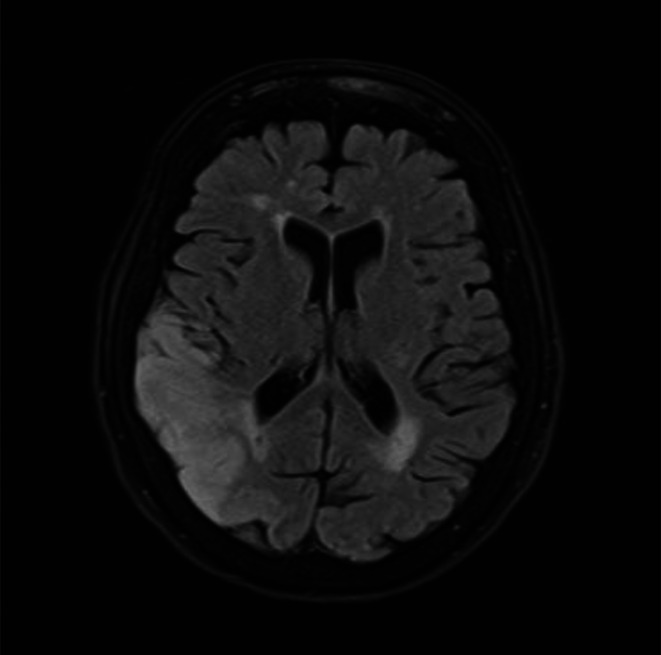
MRI brain demonstrates a large ischemic stroke in the right middle cerebral artery (MCA) territory (arrow), along with smaller ischemic lesions in the bilateral frontal lobes and the left occipital lobe (line arrows).

The anterior mediastinal lesions (measuring 1.0 and 1.2 cm) and the 1.9 × 1.8 cm right upper lobe pulmonary mass were initially concerning for septic emboli versus metastatic malignancy. Follow‐up computed tomography of the chest performed on hospital day 12 demonstrated complete resolution of both the mediastinal lesions and the pulmonary mass after antibiotic therapy. A malignancy workup, including serum tumor markers (CEA and CA 19–9) and contrast‐enhanced CT, was negative. These findings confirm that the lesions represented septic emboli secondary to *N. bacilliformis* infective endocarditis rather than malignancy.

Over the subsequent days, she remained afebrile for more than 48 h, with gradual resolution of leukocytosis and no progression of neurological deficits. The treatment course resulted in clinical stabilization and favorable outcomes, consistent with previously reported cases of *N. bacilliformis* endocarditis.

## 5 Discussion

Infective endocarditis (IE) is one of the most life‐threatening conditions with high morbidity and mortality rates despite advances in antimicrobial therapy and cardiac imaging. Large contemporary cohorts, including the International Collaboration on Endocarditis (ICE) studies, have demonstrated that elderly patients more frequently present with atypical features with neurological and embolic complications [5]. Culture‐negative infective endocarditis (BCNE) accounts for approximately 5%–10% of IE published cases, depending on geographic region and diagnostic methodology [1]. *Neisseria bacilliformis* , first described in 2006, is a rare rod‐shaped Neisseria species distinct from *Neisseria gonorrhoeae* and *Neisseria meningitidis* , and is capable of causing invasive disease. To date, fewer than five prior cases of endocarditis due to *N. bacilliformis* have been reported in the literature [4, 6, 7]. The case represents an additional rare presentation and expands the clinical spectrum of this organism.

The pathogenesis of *N. bacilliformis* IE is not fully elucidated, but proposed mechanisms are similar to those of other oral flora‐associated endocarditis pathogens. Endothelial injury leads to platelet‐fibrin deposition, creating a nidus for microbial colonization during transient bacteremia. *N. bacilliformis* being part of normal oropharyngeal flora, may enter the bloodstream following mucosal disruption. Its clinical behavior resembles HACEK organisms, which are fastidious gram‐negative bacteria associated with subacute endocarditis [6].

Transient bacteremia following mucosal disruption may allow bacterial adherence to damaged endothelium or preexisting valvular abnormalities [8]. Although the patient had no documented structural valve disease, advanced age and endothelial dysfunction associated with hypertension may have predisposed her to valvular colonization. Once bacteremia occurs, adherence to damaged endocardial surfaces can lead to vegetation formation. Structural valvular abnormalities, in particular, provide a surface conducive to biofilm formation, increasing the chances of infection. Embolization results from fragmentation of friable vegetation, which explains the systemic infarctions observed in our patient [9].

Our patient presented with abdominal pain and was found to have splenic, colic, and renal infarctions, later complicated by an ischemic stroke. Notably, she was initially afebrile and hemodynamically stable, showing an atypical presentation of fastidious organisms.

The previously reported cases of *N. bacilliformis* IE followed a similar subacute course and had favorable outcomes. One involved a bicuspid aortic valve and was treated with one month of antibiotics [4], whereas another needed valve replacement followed by six weeks of therapy [6]. Similarly, one case involved prosthetic valves and was treated with a six week course of antibiotics [7]. Similar to these reports, the patient exhibited embolic complications, reinforcing the embolic potential of this organism. Importantly, our case also demonstrated pulmonary involvement, which has not been highlighted in previously reported cases. Computed tomography angiography identified two anterior mediastinal lesions and an upper lobe pulmonary lesion pointing towards metastatic malignancy vs. embolic phenomenon. Moreover, our patient also developed a large right middle cerebral artery (MCA) territory ischemic stroke with watershed infarcts, which highlight the neurological complications of *N. bacilliformis* IE.

Diagnosis in this case relied on multimodal evaluation. Transesophageal echocardiography (TEE) demonstrated a mitral valve vegetation fulfilling a major Duke criterion. However, blood cultures remained negative. An important diagnostic consideration in contemporary IE evaluation is the role of positron emission tomography/computed tomography (PET/CT) [10]. Although PET/CT was not the primary diagnostic modality in our case, its importance in cases with suspected extracardial involvement or inconclusive echocardiographic is highly relevant. Definitive microbiological diagnosis was done using plasma microbial cell‐free DNA testing, which confirmed the presence of *N. bacilliformis* [11]. McfDNA sequencing, unlike serology testing provides broad‐range pathogen detection without requiring viable organisms, making it valuable after antibiotic exposure.

There are no standardized treatment guidelines specifically for *N. bacilliformis* endocarditis due to limited number of reported cases. Empiric intravenous broad spectrum antibiotics were initiated, in accordance with guideline recommendations. Ceftriaxone 2 g daily was continued to complete a six‐week course. It resulted in clinical stabilization and favorable outcomes, consistent with previously reported cases of *N. bacilliformis* endocarditis.

Prognosis in IE depends on patient age, comorbidities, embolic complications, and timely initiation of targeted therapy. Although neurological embolism increases the morbidity, early diagnosis and organism directed treatment improve outcomes. Preventive strategies include appropriate dental hygiene, careful management of transient bacteremia, and adherence to prophylaxis recommendations in selected high‐risk patients. To our knowledge, this case represents the fourth documented instance of *N. bacilliformis* endocarditis. It highlights the importance of considering rare Neisseria species in BCNE and recognizes the evolving role of PET/CT in its diagnosis.

This case highlights several important learning points. *Neisseria bacilliformis* represents a rare but important cause of culture‐negative infective endocarditis that carries a high risk of multisystem embolic complications involving the spleen, kidney, bowel, lungs, and brain. Plasma microbial cell‐free DNA testing serves as a valuable diagnostic modality in blood culture‐negative cases, particularly following antibiotic administration, and facilitates timely targeted therapy. Elderly patients may present with atypical features such as initial absence of fever, underscoring the need for a high index of suspicion and use of multimodal imaging combined with advanced molecular diagnostics to achieve early diagnosis and improve clinical outcomes despite significant embolic events.

## Author Contributions

**Syed Muhammad Salman Hassan:** data curation, writing – original draft. **Hareem Saleem:** conceptualization, data curation, writing – review and editing, writing – original draft. **Muhammad Saaim:** formal analysis, writing – original draft. **Anum Faiz:** supervision, writing – review and editing. **Bilal Amjad:** investigation, writing – original draft. **Ian Vargas:** writing – review and editing. **Moosa Mubarika:** writing – review and editing. **Tayyaba Saleem:** project administration, writing – review and editing. **Esha Umair:** writing – original draft, visualization. **Lima Dalil:** supervision, writing – review and editing, project administration. **Muneeb Khawar:** writing – review and editing.

## Funding

The authors have nothing to report.

## Ethics Statement

Ethics approval was not required for this case report, as it involves anonymized retrospective data from a single patient and adheres to institutional guidelines for case reporting. No identifiable patient information is included beyond what is necessary for the scientific description.

## Consent

Written informed consent was obtained from the patient for publication of this case report and any accompanying images.

## Conflicts of Interest

The authors declare no conflicts of interest.

## Data Availability

Data sharing is not applicable to this article as no new data were created or analyzed in this case report. All relevant patient data are presented within the manuscript.
